# Nitrate Transport and Distribution in Soybean Plants With Dual-Root Systems

**DOI:** 10.3389/fpls.2021.661054

**Published:** 2021-05-20

**Authors:** Sha Li, Fengsheng Xiao, Daocheng Yang, Xiaochen Lyu, Chunmei Ma, Shoukun Dong, Chao Yan, Zhenping Gong

**Affiliations:** ^1^College of Agriculture, Northeast Agricultural University, Harbin, China; ^2^College of Resources and Environment, Northeast Agricultural University, Harbin, China

**Keywords:** dual-root soybean, nitrate, transport, distribution, xylem, phloem

## Abstract

Nitrate absorbed by soybean (*Glycine max* L. Merr.) roots from the soil can promote plant growth, while nitrate transported to nodules inhibits nodulation and nodule nitrogen fixation activity. The aim of this study was to provide new insights into the inhibition of nodule nitrogen (N) fixation by characterizing the transport and distribution of nitrate in soybean plants. In this research, pot culture experiments were conducted using a dual root system of soybeans. In the first experiment, the distribution of ^15^N derived from nitrate was observed. In the second experiment, nitrate was supplied–withdrawal–resupplied to one side of dual-root system for nine consecutive days, and the other side was supplied with N-free solution. Nitrate contents in leaves, stems, petioles, the basal root of pealed skin and woody part at the grafting site were measured. Nitrate transport and distribution in soybean were analyzed combining the results of two experiments. The results showed that nitrate supplied to the N-supply side of the dual-root system was transported to the shoots immediately through the basal root pealed skin (the main transport route was via the phloem) and woody part (transport was chiefly related to the xylem). There was a transient storage of nitrate in the stems. After the distribution of nitrate, a proportion of the nitrate absorbed by the roots on the N-supply side was translocated to the roots and nodules on the N-free side with a combination of the basal root pealed skin and woody part. In conclusion, the basal root pealed skin and woody part are the main transport routes for nitrate up and down in soybean plants. Nitrate absorbed by roots can be transported to the shoots and then retranslocated to the roots again. The transport flux of nitrate to the N-free side was regulated by transient storage of nitrate in the stems.

## Introduction

Nitrogen (N) is an important macronutrient required for crop growth (Nacry et al., 2013; Obrien et al., 2016), and its utilization and transport are directly related to crop growth and yield. Soybean (*Glycine max* L. Merr.) plants can form root nodules and utilize atmospheric N_2_ in association with rhizobia, which are N fixing soil bacteria (Ohyama et al., 2018), while the addition of exogenous N source (such as NO_3_–N) is also necessary for the best seed yield. N in nitrate, as an agricultural supplement to N, is easily absorbed by crops, which can accelerate crop growth (Martinoia et al., 1981; Forde, 2002; De Angeli et al., 2006; Wang et al., 2012; Vidal et al., 2020), and nitrate transport and assimilation in soybean directly affect plant growth (Yamashita et al., 2019). The nitrate transformation ability of soybean roots is very high, and 5 mM nitrate can significantly increase nitrate transport in soybean roots (Ishikawa et al., 2018). In the absence of other exogenous N source, nitrate can induce further transcription of nitrate transporter *GmNRT2* in soybean roots, thereby increasing root uptake and transport of nitrate in soil (Amarasinghe et al., 1998). Nitrate absorbed by soybean roots is transported to the shoots through the xylem by transpiration, where it is reduced to nitrite, and then reduced to ammonium. Finally, ammonium N is assimilated through amino acids and proteins in the leaves by GS/GOGAT (glutamine synthetase/glutamate synthetase) pathways; and then, amino acids are transported downward (Yoneyama and Ishizuka, 1982; Becana and Sprent, 1987; Ohyama et al., 2017). Ohyama (1984) fed ^15^NO_3_^–^ to soybean roots and found that despite the high capacity of the roots to transform nitrate, a small proportion of ^15^NO_3_^–^ was directly translocated to the shoot. An inactive nitrate storage pool has also been speculated to exist in the stems. A short-term supply of 6.4 mM nitrate promoted N metabolism in the roots but had a pronounced inhibitory effect on nitrate metabolism in nodules (Streeter, 1985).

To better understand the transport and distribution of N in leguminous plants, split-root and dual-root experiments were performed. Supplying nitrate to one part of the roots of split-root soybeans and recycling N in the whole plant can accelerate N transport to all organs of soybeans (Tanaka et al., 1985; Li et al., 2014). Using a dual-root system in soybean, Lyu et al. (2020) added a nutrient solution with different concentrations of ^15^NH_4_^15^NO_3_ to one side of the root system, and a higher concentration of N on the N-supplied side led to a higher ^15^N level in the roots on the other side, indicating that N can be transported and distributed in soybean plants. Zhang et al. (2020) added 50 mg L^–1^ ^15^NO_3_^–^ or ^15^NH_4_^+^ to one side of the dual-root system of soybean and found that after the ^15^N from the two N sources was transported to the shoot, it was redistributed to the roots and nodules.

High concentrations of nitrate inhibit nodulation and N fixation by root nodules in soybean (Streeter, 1982; Fujikake et al., 2003; Gan et al., 2004; Mbah and Dakora, 2018; Du et al., 2020). Supplying 10 mM nitrate to soybeans for 48 h increased the relative growth rate of the whole plant and inhibited nodule growth (Vessey et al., 1988). Fujikake et al. (2002) presumed that both soybean nodule growth and N_2_ fixation activity were adjusted by sensing of the nitrate concentration in roots and nodules. Xia et al. (2017) supplied nitrate to one side of a dual-root system of soybean and noted that the nodule number and nitrogenase activity were strongly inhibited in the roots on the side with no N supply, indicating that nitrate transport and distribution can affect N fixation by nodules.

Direct application of nitrate to soybean roots can be a good approach to study the distribution of nitrate and its assimilates in plants (Bacanamwo and Harper, 1997; Sato et al., 1999), and in this case, nitrate demonstrated only to be transported from the roots to the shoots. The split-root plant system consists of splitting the root into two parts such that neither of the two subsystems maintains integrity. Use of the split-root method only confirmed N transport (Ohyama et al., 2018), and whether the nitrate transported to the shoots will be transported to the roots again is not clear yet. The aim of this study was to characterize the transport and distribution of nitrate in soybean plants. We supplied nitrate to one side of a dual-root system of soybean and changed the concentration of N supplied over different experimental phases. By combining this strategy with the ^15^N tracer technique, we carried out a systematic study on nitrate content change and distribution in various organs of soybeans.

## Materials and Methods

In 2017 and 2018, this study was carried out in the Experimental Base of Northeast Agricultural University located in Xiangfang District, Harbin, Heilongjiang Province, China (geographical coordinates: 126°43′E, 45°44′N). Soybean plants with dual-root systems were prepared according to Xia et al. (2017). The soybean seeds (*Glycine max* L. cv. Dongda1) were seeded into fine-sand medium and cultured in an illuminated growth chamber at 30°C for approximately 3 days. Two seedlings were selected and grafted into a dual-root system, and each pot contained two plants with dual-root systems (see Supplementary Figure 1 for the detailed method). N-free nutrient solution was prepared according to the description provided by Hoagland and Arnon (1950) and Dong et al. (2010); the nutrient solution ingredients are listed in Supplementary Table 2. During the VC stage (unfolded cotyledon stage), field-grown soybean nodules that were frozen in the previous year were ground and added to the nutrient solution, with approximately 5 g/L for *Rhizobium* inoculation, and inoculated for five consecutive days. Before the VC stage, the plants were irrigated with distilled water once a day; from the VC stage to the V4 stage (fourth trifoliate leaf stage), the plants were irrigated with the nutrient solution once a day; and from the V4 stage to the end of the experiment, the plants were irrigated with the nutrient solution twice a day in the morning and evening. The irrigation volume was 250 mL on each side of the root system, and the N source was KNO_3_. While KNO_3_ was added to one side (N+), K_2_SO_4_ was used to replace KNO_3_ on the other side (N–) and in the control treatment to ensure an equal concentration of K^+^ between the treatments. Stages were designated according to the description of Fehr et al. (1971).

### Experimental Treatments

#### ^15^N Labeling Experiment

In 2017, an ^15^N labeling experiment was conducted during the VC stage to the R1 stage (initial flowering stage) of soybeans with dual-root systems. The experiment was a completely random design with three biological replications. In the N50 treatment, N was supplied to one side of the root system after 7 days of grafting. The N+ side was treated with a nutrient solution with a N concentration of 50 mg L^–1^ K^15^NO_3_ (3.63 atom% ^15^N), while the N– side was treated with the N-free nutrient solution. In the N0 treatment (control), both sides were treated with the N-free nutrient solution. The experiment was ended in the R1 stage (42 days after grafting).

#### Nitrate Content Change Experiment

In 2018, a nitrate content change experiment was conducted during the VC–R1 stages of soybeans with dual-root systems. Between the VC and V4 stages, the experimental materials were irrigated with a nutrient solution with a N concentration of 25 mg L^–1^ KNO_3_ on both sides of the root system. In the V4 stage, the N-free nutrient solution was added to both sides for N starvation over 10 days. After N starvation, in the R1 stage (42 days after grafting), the experiment was carried out over three consecutive phases, and the treatments in each phase were performed for 3 days (cumulative treatment duration: 9 days). The experiment included four treatments, designated N_*TTT*_, N_*HHH*_, N_*HTT*_, and N_*HTH*_. (1) N_*HHH*_ :the N+ side was treated with a nutrient solution with a N concentration of 100 mg L^–1^ KNO_3_ in phase I, II, and III, respectively; (2) N_*HTT*_ :the N+ side was treated with a nutrient solution with a N concentration of 100 mg L^–1^ KNO_3_ in phase I and treated with the N-free nutrient solution in phase II and III, respectively. (3) N_*HTH*_: The N+ side was treated with a nutrient solution with a N concentration of 100 mg L^–1^ KNO_3_ in phase I and resupplied 100 mg L^–1^ KNO_3_ in phase III after treating with N-free nutrient solution in phase II. N- side of the above three treatments were treated with the N-free nutrient solution. (4) N_*TTT*_: both sides of the root system were treated with the N-free nutrient solution as control. The experiment was a completely random design with three biological replications. The concentrations of treatments on the N+ side and the four treatments in which phase sampling was performed in Table 1.

**TABLE 1:** The concentration of N supplied to the N+ side of the dual-root soybean and the sampling phase in nitrate content change experiment.

**Treatments**	**Phase I**	**Phase II**	**Phase III**
N_*TTT*_	0 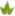	0 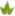	0 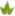
N_*HHH*_	100 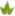	100 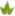	100 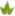
N_*HTT*_	100	0 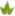	0 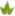
N_*HTH*_	100	0	100 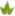

*There had three serial phases in this experiment, each phase performed for 3 days from 42 to 51 days after grafting. 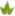 represented this treatment was sampling in this phase. 0 or 100 represented the concentration of N supplied to the N+ side of the dual-root soybean, the unit was mg L^–1^. N- side of the dual-root system were treated with N-free nutrient solution in the four treatments during the three phases.*

### Sampling Methods

At the end of the ^15^N labeling experiment, soybean plants with dual-root systems were cut at the grafting site. The shoots were divided into leaves, stems, and petioles, and the underground parts were divided into roots and nodules on the N+ and N– sides, respectively. All samples were washed with distilled water to remove sand and blot-dried with filter paper. The samples were then oven-dried at 65°C and used for determination of the ^15^N abundances in each plant part.

At the end of each phase of the nitrate content change experiment, soybean plants with dual-root systems were cut at the grafting site. The shoots were divided into leaves, stems, and petioles, and the underground parts were divided into roots and nodules on the N+ and N– sides, respectively. The basal parts of the roots on both sides were cut at approximately 2 cm from the grafting site and divided into pealed skin (including epidermis, cortex, and phloem, phloem was the main transport route) and woody part (including xylem and pith, xylem was the main transport route; see Supplementary Figure 3 for the sampling methods). All samples were washed with distilled water and blot-dried with filter paper. three pots of soybeans measured nodule nitrogenase activity immediately, three other pots of soybeans then frozen in liquid N_2_ and stored in a –80°C refrigerator until nitrate content measurement in each plant part.

### Analytical Methods

^15^N abundances assay: Sample solutions prepared by the Kjeldahl method were concentrated and allowed to react with lithium hypobromite under freezing-vacuum conditions to produce N_2_. ^15^N abundances (atom %) was determined by a dual-inlet mass spectrometer (Delta V Advantage IRMS; Thermo Fisher Scientific, Wilmington, DE, United States).

#### Plant Nitrate Content Assay

The nitrate content in various plant organs was determined following the method of Cataldo et al. (1975) with slight modifications. To improve the extraction rate and reduce interference from colored substances, the extraction temperature and centrifugation rate were increased. Briefly, the samples were extracted at 90°C for 1 h and then centrifuged at 15,000 × *g* for 15 min. The supernatants were allowed to react with concentrated sulfuric acid–salicylic acid solution for 30 min. Then, NaOH was added for 5 min of color development. The absorbance of the reaction mixtures at 410 nm was measured in a microplate reader (ELx800; Bio-Tek Inc., Doraville, GA, United States).

#### Nodule Nitrogenase Activity

Acetylene reduction method described in Gremaud and Harper (1989) was used to measure nitrogenase activity in root nodules.

### Data Analysis

All statistical analyses were performed with IBM SPSS 21.0 (IBM Corp., Armonk, NY, United States). All data were tested for normality before one-way analysis of variance, and Duncan’s multiple range test was run for mean comparisons at a significance level of *p* < 0.05.

## Results

### ^15^N Abundance (Atom %) and N Source of Soybean Plants With Dual-Root Systems

Table 2 shows the ^15^N abundances in various organs of soybean plants grown in sand culture under long-term exposure to nitrate containing nutrient solution (N concentration: 50 mg⋅L^–1^) supplying 3.63 atom % ^15^N to one side of the dual-root system. In the N0 treatment with no nitrate supply, natural ^15^N abundances were detected in the leaves, stems, petioles, roots, and nodules. In the N50 treatment with nitrate supply, the ^15^N abundances in the leaves, stems, and petioles as well as the roots and nodules on the N+ and N– sides were significantly (*p* < 0.05) higher than those in the N0 treatment. The ^15^N abundances in various organs of soybean plants under the N50 treatment were always higher than those under the N0 treatment (natural abundance levels) but were lower than the ^15^N abundances in the nutrient solution (3.63 atom%), indicating that the plant N was derived from the N fixed by nodules and the N contained in the nutrient solution. In the N50 treatment, although the roots and nodules on the N– side did not directly absorb ^15^NO_3_^–^ from the nutrient solution, their ^15^N abundances were significantly higher than those in the N0 treatment, showing that the ^15^N absorbed from the N+ side was translocated to the roots and nodules on the N– side through the shoots of soybean plants.

**TABLE 2:** ^15^N abundance (atom%) in the organs of soybean plants with dual-root systems.

**Treatments**	**Leaves**	**Stems**	**Petioles**	**Roots**		**Nodules**	
				**N+**	**N−**	**N+**	**N−**
N0	0.40 ± 0.010^b^	0.43 ± 0.020^b^	0.40 ± 0.009^b^	0.39 ± 0.003^b^	0.39 ± 0.003^b^	0.37 ± 0.000^b^	0.37 ± 0.000^b^
N50	1.37 ± 0.052^a^	1.27 ± 0.008^a^	1.32 ± 0.038^a^	2.21 ± 0.060^a^	0.97 ± 0.022^a^	0.78 ± 0.013^a^	0.60 ± 0.007^a^

*In the N0 treatment, an N-free nutrient solution was added to both the N+ and N– sides; in the N50 treatment, a nutrient solution with the N concentration of 50 mg L^–1^ K^15^NO_3_ was added to the N+ side, and an N-free nutrient solution was added to the N– side. Values are means ± standard error (*n* = 3). Different lowercase letters indicate a significant difference between the treatments at the 5% level by longitudinal comparison.*

Based on the ^15^N abundances of soybean plants with dual-root systems (Table 2), we calculated the percentages of N derived from nitrate in the nutrient solution (N_*NO3*_^–^) and the N fixed by nodules (N_*nodule*_) in various organs under the N50 treatment (Table 3). The roots on the N+ side had significantly higher N_*NO3*_^–^% than the other organs. No significant differences in N_*NO3*_^–^% were found between the leaves, stems, and petioles, but the N_*NO3*_^–^% values of these three organs were significantly lower than those of the roots on the N+ side and higher than those of the nodules on the N+ side as well as the roots and nodules on the N– side. These results suggested that the nitrate absorbed by the roots on the N+ side was mainly transported to the organs in the shoots. Additionally, a proportion of ^15^N transported to the leaves, stems, and petioles was distributed to the roots and nodules on the N– side. The N_*NO3*_^–^% and N_*nodule*_% were 18.53 and 81.47% in the roots on the N– side and 7.20 and 92.8% in the nodules on the same side, respectively. Since the roots and nodules on the N– side were not directly exposed to nitrate in the nutrient solution, these results indicated that 18.53 and 7.20% of the N in the roots and nodules on the N– side was translocated through the shoots from the nitrate absorbed by the roots on the N+ side, respectively.

**TABLE 3:** The percentage of N sources in soybean plants under the N50 treatment (%).

**Organs**	**Nitrogen treatments**	***N*_*NO3*_^–^**	***N*_*nodule*_**
Leaves		30.69 ± 1.58^*b*^	69.31 ± 1.58^*d*^
Stems		27.85 ± 0.25^*b*^	72.15 ± 0.25^*d*^
Petioles		29.34 ± 1.18^*b*^	70.66 ± 1.18^*d*^
Roots	N+	56.57 ± 1.83^*a*^	43.43 ± 1.83^*e*^
	N-	18.53 ± 0.66^*c*^	81.47 ± 0.66^*c*^
Nodules	N+	12.81 ± 0.41^*d*^	87.19 ± 0.41^*b*^
	N–	7.20 ± 0.23^*e*^	92.80 ± 0.23^*a*^

*N_*NO3*_^–^ is the N derived from K^15^NO_3_ in the nutrient solution, and N_*nodule*_ is the N fixed by root nodules. Values are means ± standard error (*n* = 3). Different lowercase letters indicate a significant difference in the same parameter between various organs of soybean plants at the 5% level by longitudinal comparison.*

Based on the total nitrogen (N_*all*_) accumulation in various organs of soybean plants under the N50 treatment and the percentages of N derived from nitrate in the nutrient solution data (Table 3), we calculated N_*all*_ and N_*NO3*_^–^ accumulation in various organs and the percentages of their accumulation among the whole-plant total N and nitrate accumulation [Organ/plant (%)], respectively (Table 4). With regard to the distribution ratio, the Organ/plant (%) values for both N_*all*_ and N_*NO3*_^–^ in the shoots were greater than 80%, and the Organ/plant (%) values of leaves, stems, and petioles for N_*all*_ and N_*NO3*_^–^ were consistent, indicating that the nitrate absorbed by the roots on the N+ side was also mainly distributed to the leaves, stems, and petioles. The roots on the N+ side had significantly higher N_*NO3*_^–^ accumulation than the roots on the N– side, while the nodules on the N+ and N– sides had similar N_*NO3*_^–^ accumulation. This result further indicated that the nitrate absorbed from the N+ side was primarily transported to the shoots, and more nitrate had accumulated in the roots on the N+ side than on the N– side. The Organ/plant (%) for N_*NO3*_^–^ on the N– side was 3.96%, divided into 2.73% in the roots and 1.23% in the nodules. This result showed that 2.73 and 1.23% of the nitrate absorbed by the roots on the N+ side was translocated through the shoots to the roots and nodules on the N– side, respectively.

**TABLE 4:** N accumulation and distribution in soybean plants under the N50 treatment.

**Organs**	**Nitrogen treatments**	**Nitrogen accumulation(mg plant^–1^)**		**Organ/Plant(%)**	
		**N_*all*_**	**N_*NO3*_^–^**	**N_*all*_**	**N_*NO3*_^–^**
Leaves		139.20 ± 17.29	42.22 ± 3.26	59.15 ± 7.35	61.58 ± 4.76
Stems		36.57 ± 5.86	10.16 ± 1.54	15.54 ± 2.49	14.81 ± 2.25
Petioles		17.55 ± 2.84	5.08 ± 0.62	7.46 ± 1.21	7.41 ± 0.91
Shoots total		193.32	57.46	82.15	83.80
Roots	N+	13.39 ± 1.23	7.54 ± 0.54*	5.69 ± 0.52	11.00 ± 0.78*
	N–	10.24 ± 1.93	1.87 ± 0.28	4.35 ± 0.82	2.73 ± 0.41
Nodules	N+	6.62 ± 0.58*	0.84 ± 0.06	2.81 ± 0.25*	1.23 ± 0.08
	N–	11.77 ± 0.96	0.84 ± 0.04	5.00 ± 0.41	1.23 ± 0.06
Roots total		42.02	11.09	17.85	16.20
Plants total		235.34	68.55	100.00	100.00

*N*_*all*_ is total nitrogen, including NO_3_^–^-N and nodule-fixed N, other descriptions see Table 3. Organ/plant means the percentage of the nitrate accumulation in various organs accumulation out of the whole-plant nitrate accumulation. Values are means ± standard error (*n* = 3 *Indicates a significant difference in the same parameter of the roots or nodules between the N+ and N– sides at the 5% level by longitudinal comparison.

### Nitrate Content of Soybean Plants With Dual-Root Systems

#### Nitrate Content of Stems and Petioles

During the three phases of the experiment, changing the nitrate concentration of the nutrient solution supplied to the N+ side had a significant effect on the nitrate contents in the stems and petioles of soybean plants with dual-root systems (Table 5). At phases I, II, and III, the nitrate contents of the stems and petioles were significantly higher in the N_*HHH*_ treatment than the N_*TTT*_ treatment, indicating that the supply of 100 mg L^–1^ NO_3_^–^-N to one side of the dual-root system of soybeans significantly increased the nitrate contents in the stems and petioles. As the experimental phase progressed, the nitrate contents of the stems and petioles showed an increasing trend in the N_*HHH*_ treatment.

**TABLE 5:** Nitrate content in the leaves, stems, and petioles of soybeans (μg g^–1^ FW).

**Organs**	**Treatments**	**Nitrogen Concentration (mg L^–1^)**	**Phase I**	**Phase II**	**Phase III**
Stems	N_*TTT*_	0–0–0	88.9 ± 39.18^*b*^	58.4 ± 7.99^*c*^	168.5 ± 3.45^*b*^
	N_*HHH*_	100–100–100	242.8 ± 22.15^*a*^	445.3 ± 4.79^*a*^	748.0 ± 50.41^*a*^
	N_*HTT*_	100–0–0		176.8 ± 18.41^*b*^	194.4 ± 39.26^*b*^
	N_*HTH*_	100–0–100			262.2 ± 35.94^*b*^
Petioles	N_*TTT*_	0–0–0	89.7 ± 8.31^*b*^	113.6 ± 7.18^*c*^	38.8 ± 1.70^*d*^
	N_*HHH*_	100–100–100	403.2 ± 29.09^*a*^	538.9 ± 25.38^*a*^	840.2 ± 12.58^*a*^
	N_*HTT*_	100–0–0		303.2 ± 16.02^*b*^	272.8 ± 7.66^*c*^
	N_*HTH*_	100–0–100			670.8 ± 40.54^*b*^

*The treatments and N concentrations are described Table 1. Values are means ± standard error (*n* = 3). Different lowercase letters indicate a significant difference between the treatments at the 5% level (Duncan’s test) by longitudinal comparison.*

At the end of phase II, the nitrate contents of the stems and petioles across the three treatments were ranked in the following order: N_*HHH*_ > N_*HTT*_ > N_*TTT*_. The differences between the treatments all reached the level of significance. At phase III, the same order of nitrate contents was maintained across the three treatments, although the difference in the nitrate contents of the stems was not significant between N_*HTT*_ and N_*TTT*_. In the N_*HTT*_ treatment, the nitrate concentration of the nutrient solution supplied to the N+ side was changed to 0 mg L^–1^ from 100 mg L^–1^ after phase I, and the plants were continuously cultured throughout phases II and III, resulting in a prominent decrease in the nitrate contents of the stems and petioles. This result indicated that the concentration of N supplied to the N+ side affected the nitrate contents of the stems and petioles. This significant decrease in nitrate in the stems and petioles after N supply and withdrawal indicates that the nitrate absorbed by the roots on the N+ side was directly transported to the shoots.

At the end of phase III, the nitrate content of the petioles in the N_*HTH*_ treatment was significantly higher than those in the N_*TTT*_ and N_*HTT*_ treatments but significantly lower than that in the N_*HHH*_ treatment, showing that after N supply–N withdrawal–N resupply, the nitrate contents of the petioles also exhibited a trend of increase–decrease–increase. The nitrate contents of the stems did not differ significantly between N_*HTH*_ and N_*TTT*_ but exhibited a trend similar to that in the petioles. In the N_*HTH*_ treatment of phase III, the nitrate content of the stems was similar to the value obtained with the N_*HHH*_ treatment in phase I but was only one-third of the value obtained with the N_*HHH*_ treatment in phase III. This result indicated that if N was continuously supplied to soybean plants, nitrate could accumulate in the stems, while if N was withdrawn and then resupplied, the nitrate content of the stems would not remain high, showing that the nitrate storage in the stems was transient.

#### Nitrate Content of Roots and Nodules

High concentrations of nitrate inhibited nodule nitrogenase activity regardless of whether nodules were directly exposed to nitrate or not (for the data, see Supplementary Table 5). The results in Table 6 show the effect of changing the nitrate concentration of the nutrient solution on the nitrate contents in the roots and nodules on both sides of soybean plants with dual-root systems during the experiment. At phases I, II, and III, the nitrate contents of the roots and nodules on the N+ and N– sides were significantly higher in the N_*HHH*_ treatment than the N_*TTT*_ treatment. Additionally, the nitrate contents of the roots and nodules on the N+ side were significantly higher than those on the N– side in all three phases. This result indicated that the supply of 100 mg L^–1^ NO_3_^–^-N to one side of the dual-root system of soybeans considerably increased the nitrate contents of the roots and nodules on both the N+ and N– sides. The roots on the N+ side were directly exposed to nitrate, leading to a substantially higher nitrate content in the roots and nodules on the N+ side than on the N– side. Despite no direct exposure to nitrate on the N– side, a proportion of nitrate was still translocated to this side through the shoots.

**TABLE 6:** Nitrate content in the roots and nodules of soybeans (μg g^–1^ FW).

**Treatments**		**Nitrogen concentration (mg L^–1^)**	**Phase I**		**Phase II**		**Phase III**	
			**N+**	**N–**	**N+**	**N–**	**N+**	**N–**
Roots	N_*TTT*_	0–0–0	32.2 ± 1.09^*b*^	32.2 ± 1.09^*b*^	39.6 ± 7.17^*c*^	39.6 ± 7.17^*b*^	59.1 ± 1.88^*d*^	59.1 ± 1.88^*d*^
	N_*HHH*_	100–100–100	713.3 ± 28.47^a^*	171.5 ± 11.09^*a*^	681.5 ± 46.31^a^*	180.5 ± 9.73^*a*^	699.7 ± 10.26^a^*	143.6 ± 5.14^*b*^
	N_*HTT*_	100–0–0			227.3 ± 1.88^b^*	189.6 ± 1.44^*a*^	159.6 ± 11.63^c^*	99.4 ± 11.26^*c*^
	N_*HTH*_	100–0–100					478.2 ± 9.63^b^*	171.6 ± 5.35^*a*^
Nodules	N_*TTT*_	0–0–0	34.8 ± 1.29^*b*^	34.8 ± 1.29^*b*^	40.2 ± 3.28^*c*^	40.2 ± 3.28^*b*^	46.4 ± 0.65^*c*^	46.4 ± 0.65^*d*^
	N_*HHH*_	100–100–100	242.3 ± 13.50^a^*	67.8 ± 2.53^*a*^	222.1 ± 4.72^a^*	74.3 ± 8.49^*a*^	152.7 ± 14.40^a^*	65.5 ± 1.05^*b*^
	N_*HTT*_	100–0–0			113.6 ± 6.22^b^*	71.5 ± 0.16^*a*^	90.3 ± 0.71^b^*	58.2 ± 0.88^*c*^
	N_*HTH*_	100–0–100					155.9 ± 16.90^a^*	77.5 ± 0.87^*a*^

The treatments and N concentrations are described in Table 1. Values are means ± standard error (*n* = 3). Different lowercase letters indicate a significant difference between the treatments at the 5% level (Duncan’s test) by longitudinal comparison. * Indicates a significant difference between the N+ and N– sides under the same treatment at the 5% level by horizontal comparison.

At the end of phase II, the nitrate contents of the roots and nodules on the N+ side under the three treatments were ranked as N_*HHH*_ > N_*HTT*_ > N_*TTT*_, and the differences between the treatments were significant. At phase III, the same order of nitrate contents was still seen across the three treatments, and the differences between the treatments were still significant. These results showed that the nitrate contents of the roots and nodules on the N+ side increased with the addition of nitrate from the nutrient solution, while they decreased rapidly with the withdrawal of nitrate. At phase II, consistent changes in the nitrate contents of the roots and nodules were observed on the N– side, and the difference between N_*HTT*_ and N_*HHH*_ was not significant, but the nitrate contents in these two treatments were significantly higher than those in the N_*TTT*_ treatment. At phase III, the nitrate contents of the roots and nodules on the N– side were both ranked N_*HHH*_ > N_*HTT*_ > N_*TTT*_, and the differences between the treatments were significant. This result indicated that after N withdrawal, the decrease in the nitrate contents of the roots and nodules on the N– side was delayed without direct exposure to nitrate. During phase II, N was withdrawn in the N_*HTT*_ treatment only for 3 days, and the resulting nitrate contents of the roots on the N– side did not decrease but were slightly higher than those in the N_*HHH*_ treatment during the same phase. However, at phase III, the nitrate contents of the roots on the N– side decreased significantly in the N_*HTT*_ treatment compared with the N_*HHH*_ treatment, and the same trend of changes was observed in the nodules on the N– side. Nevertheless, at phase II, the nitrate contents of the nodules on the N– side were not significantly higher in the N_*HTT*_ treatment than in the N_*HHH*_ treatment during the same phase. A plausible reason is a delay in the root response to the supplied nitrate concentration, which affected nitrate absorption and metabolism by the nodules. In the N_*HTT*_ treatment in phases II and III, the nitrate contents of both the roots and nodules on the N+ side were significantly higher than those on the N– side, indicating that whether N was withdrawn for 3 or 6 days, a certain content of nitrate would accumulate in the roots and nodules on the N+ side compared with the N– side.

At the end of phase III, in the N_*HTH*_ treatment, the nitrate content of the roots on the N+ side was significantly lower than that in the N_*HHH*_ treatment and higher than those in the N_*TTT*_ and N_*HTT*_ treatments. This result showed that after N supply–N withdrawal–N resupply, the nitrate content of the roots on the N+ side increased again with the addition of nitrate to the nutrient solution, but this increased nitrate content was considerably lower than that in the N_*HHH*_ treatment (under long-term N supply). The nodules on the N+ side showed similar changes as the root nitrate content on the same side, although the difference between N_*HTH*_ and N_*HHH*_ was not significant. This result showed that the nitrate contents of the roots and nodules on the N+ side followed a trend of increase–decrease–increase as the concentration of nitrate in the nutrient solution supplied to the same side changed. In the N_*HTH*_ treatment, the nitrate contents of the roots and nodules on the N– side were significantly higher than those in the other three treatments, indicating that the roots and nodules on the N– side were sensitive to changes in the nitrate concentration of the nutrient solution.

#### Nitrate Content of the Basal Root Pealed Skin and Woody Part

During the experiment, the effects of changing the nitrate concentration of the nutrient solution on the nitrate contents in the basal root pealed skin and woody part (Table 7) exhibited the same trend as observed in the roots and nodules of soybean plants with dual-root systems. At phases I, II, and III, the nitrate contents of the basal root pealed skin and woody part on both the N+ and N– sides were significantly higher in the N_*HHH*_ treatment than the N_*TTT*_ treatment. Additionally, the nitrate contents of the basal root pealed skin and woody part were significantly higher on the N+ side than on the N– side in all three phases. These results indicated that the supply of 100 mg L^–1^ NO_3_^–^-N to one side of the dual-root system of soybeans markedly increased the nitrate contents in the basal root pealed skin and woody part on both the N+ and N– sides.

**TABLE 7:** Nitrate content in the basal root pealed skin and woody part of soybeans (μg g^–1^ FW).

**Treatments**		**Nitrogen concentration (mg L^–1^)**	**Phase I**		**Phase II**		**Phase III**	
			**N+**	**N–**	**N+**	**N–**	**N+**	**N–**
Pealed skin	N_*TTT*_	0–0–0	69.0 ± 2.26^*b*^	69.0 ± 2.26^*b*^	64.4 ± 2.47^*c*^	64.4 ± 2.47^*b*^	73.6 ± 2.55^*c*^	73.6 ± 2.55^*b*^
	N_*HHH*_	100–100–100	592.0 ± 26.95^a^*	186.4 ± 15.38^*a*^	546.3 ± 29.84^a^*	172.1 ± 24.75^*a*^	472.8 ± 28.93^a^*	99.3 ± 6.53^*ab*^
	N_*HTT*_	100–0–0			306.6 ± 27.75^b^*	182.8 ± 10.26^*a*^	196.2 ± 13.12^b^*	103.2 ± 6.15^*ab*^
	N_*HTH*_	100–0–100					456.8 ± 4.72^a^*	119.8 ± 13.32^*a*^
Woody part	N_*TTT*_	0–0–0	14.4 ± 2.70^*b*^	14.4 ± 2.70^*b*^	15.9 ± 2.28^*c*^	15.9 ± 2.28^*b*^	19.0 ± 2.89^*c*^	19.0 ± 2.89^*b*^
	N_*HHH*_	100–100–100	86.7 ± 12.83^a^*	42.0 ± 4.23^*a*^	280.0 ± 14.93^a^*	34.4 ± 3.79^*a*^	274.1 ± 5.28^a^*	58.1 ± 3.95^*a*^
	N_*HTT*_	100–0–0			76.0 ± 1.27^b^*	30.2 ± 2.66^*a*^	53.3 ± 6.72^*b*^	50.1 ± 3.91^*a*^
	N_*HTH*_	100–0–100					272.2 ± 7.93^a^*	77.2 ± 10.93^*a*^

The treatments and N concentrations are described in Table 1. Values are means ± standard error (*n* = 3). Different lowercase letters indicate a significant difference between the treatments at the 5% level (Duncan’s test) by longitudinal comparison.*Indicates a significant difference between the N+ and N– sides under the same treatment at the 5% level by horizontal comparison.

At the end of phase II, the nitrate contents of the basal root pealed skin and woody part on the N+ side were both ranked as N_*HHH*_ > N_*HTT*_ > N_*TTT*_, and the differences between the treatments were significant. At phase III, a consistent ranking of nitrate contents of the basal root pealed skin and woody part on the N+ side was maintained across the three treatments, and the differences between the treatments were also significant. These results showed that the nitrate contents of the basal root pealed skin and woody part on the N+ side increased with the addition of nitrate to the same side and decreased with the withdrawal of nitrate. At phase II, in the N_*HTT*_ treatment, the nitrate contents of the basal root pealed skin and woody part on the N– side showed no significant difference compared with those of the N_*HHH*_ treatment, but the values were significantly higher than they were in the N_*TTT*_ treatment. At the end of phase III, in the N_*HTT*_ treatment, the nitrate contents of the basal root pealed skin on the N– side were close to the values of the N_*HHH*_ treatment and did not differ significantly from those in the N_*TTT*_ treatment. Additionally, in the N_*HTT*_ treatment, the nitrate content of the basal root woody part on the N– side was significantly higher than in the N_*TTT*_ treatment and slightly lower than that in the N_*HHH*_ treatment. These results showed that N supply–N withdraw–N withdraw to the N+ side did not reduce the nitrate content in the pealed skin or woody part on the N– side, obviously.

At the end of phase III, in the N_*HTH*_ treatment, the nitrate contents of the basal root pealed skin and woody part on the N+ side were close to the values of the N_*HHH*_ treatment and differed significantly from the N_*TTT*_ and N_*HTT*_ values. In other words, these nitrate contents increased with the addition of nitrate to the N+ side and decreased with the withdrawal of nitrate. These results showed that the nitrate absorbed by the roots was transported to the shoots through the basal root pealed skin and woody part on the N+ side. In the N_*HTH*_ treatment, the nitrate contents of the basal root pealed skin and woody part on the N– side were higher than those in the other three treatments, but the difference was significant only when compared with the N_*TTT*_ treatment. This result indicated that N supply considerably increased the nitrate contents of the basal root pealed skin and woody part on the N– side.

## Discussion

### Absorption and Translocation of Nitrate in Soybean Plants

Mcneil et al. (1979) indicated that in lupin (*Lupinus micranthus* Guss.), asparagine is the main form of N transported from the roots to the shoots. Sprent and Thomas (1984) suggested that in broad bean (*Vicia faba* L.) and pea (*Pisum sativum* L.), nitrate is assimilated in the roots and then transported to the shoots. When nitrate is supplied to soybean plants, the N derived from nitrate is mainly transported in the form of asparagine to the shoots (Ohyama, 1984) but is also directly transported in the form of nitrate to the shoots (Rufty et al., 1982; Reynolds et al., 1990). Ohyama and Kumazawa (1979) considered that the majority of the nitrate absorbed by soybean roots is transported to the shoots and assimilated in the leaves, with a proportion of nitrate being reduced in the roots. Andrews (1986) proposed that in lupin, pea, and clover (*Trifolium pratense* L.), when the concentration of nitrate supplied is 1 mol m^−3^, the nitrate is predominantly assimilated in the roots, but when the concentration of nitrate supplied is 20 mol m^−3^, the nitrate content of xylem sap increases rapidly in all three legume crops, and the nitrate is transported to the shoots for assimilation. However, in soybean and kidney bean (*Phaseolus vulgaris* Linn.), nitrate is directly transported to the shoots and assimilated there under either a low or a high concentration of nitrate supply. In this study, the ^15^N abundances of whole soybean plants were prominently increased by supplying ^15^NO_3_^–^ to one side of dual-root system (Table 2). Moreover, the nitrate contents of the stems and petioles increased with N supply to the N+ side, whereas they decreased with N withdrawal from the N+ side (Table 5). These results suggest that soybean roots can directly absorb nitrate and transport it to the stems and petioles and then redistribute it to other organs. With increasing N supply time, a certain content of nitrate can accumulate in the stems, and the accumulated nitrate may serve as transient storage (Ohyama, 1984).

After the addition of ^15^NH_4_NO_3_ to the leaves of alfalfa, most of the N is translocated to the roots and new leaves, whereas 10% of the ^15^N is detected in the root nodules (Sulieman et al., 2010). When ^15^NO_3_^–^ is added to the second fully expanded leaf blades of sunflower plants, ^15^N abundances are detected in the upper internodes and lower internodes of N-treated leaves, while the ^15^N abundances in the upper internodes are lower than those in the lower internodes (Ito and Kumazawa, 1976), indicating that the N added to the leaves can be transported upward and downward. Ohyama et al. (1989) and Sato et al. (1999) added ^13^NO_3_^–^ and ^15^NO_3_^–^ to soybean plants respectively, and found that ^13^N or ^15^N is not transported to the root nodules within a short time (1 h), showing a time lag. In this study, the roots and nodules on the N– side were not directly exposed to nitrate in the nutrient solution; however, 18.53 and 7.2% of the N that accumulated in the roots and nodules, respectively, were derived from the nitrate absorbed by the roots on the N+ side, accounting for 3.69% of the whole-plant accumulation of nitrate (Tables 3, 4). The results indicate that supplying N to one side of the dual-root system can provide N to the whole soybean plant (including nodules) (Zhang et al., 2020). The nitrate contents of the roots and nodules on the N– side increased with the addition of nitrate to the N+ side and decreased with nitrate withdrawal, suggesting that the nitrate absorbed from the N+ side can be directly translocated through the shoots to the roots and nodules on other side (N–). Krapp et al. (2014) found that a NO_3_^–^ transporter, *MtNRT2.3*, is expressed in the shoots and nodules of alfalfa, where it regulates nitrate transport throughout the plant. Additionally, Amarasinghe et al. (1998) indicated that *GmNRT2* is a high-affinity nitrate transporter involved in nitrate absorption by soybean roots, and the mRNA expression level of *GmNRT2* is considerably high in soybean roots continuously exposed to high concentrations of nitrate despite very low activity of this high-affinity nitrate transporter.

The nodules’ growth and nitrogenase activity are inhibited by N source, which is related to N concentration and application position. Researches showed that high nitrate concentrations (10 or 14 mM) inhibited growth and nitrogenase activity of nodules (Daimon et al., 1999; Gan et al., 2004; Kato et al., 2010), and low nitrate concentrations (0.7 or 3.5 mM) significantly promoted the growth and nitrogenase activity of nodules (Daimon and Yoshioka, 2001). Moreover, Yamashita et al. (2019) found that supplying 1 mM nitrate for 5 days can significantly inhibited dry weight and nitrogenase activity in soybean. Research in peanut (*Arachis hypogaea* L.) split-root system showed that the nodules weight and nitrogenase activity on the N-free side were not significantly affected if supplying nitrate to one side of the peanut split-root system for 5 days, while significantly decreased when supplying for 30 days (Daimon and Yoshioka, 2001). In this study, supplying nitrate of higher concentration to the N+ side significantly inhibited the nodule nitrogenase activity of the both side, while withdrawal of nitrate lead to a smooth increase in nodule nitrogenase activity on the both side (Supplementary Table 5). The same result was obtained in Cho and Harper’s (1991) research. The dry weight of nodules on the N- side were not affected by the nitrate transport from the shoots to this side, and the dry weight of nodules on the N+ side were affected by the nitrate in phase III (Supplementary Table 4). These results indicated that the growth and nitrogenase activity of soybean nodules were affected by nitrate concentration, N supply time and supply site; the nitrate transported to the N- side inhibited the nitrogenase activity of the nodules on this side, but did not significantly affect the growth of the nodules.

### Xylem and Phloem Are the Routes for Bidirectional Transport of Nitrate

Yoneyama and Ishizuka (1982) found evidence that nitrate is transported to the shoots through the xylem by transpiration. Additionally, nitrate translocation depended on the rates of nitrate uptake and loading into xylem (Orieux et al., 2018). In *Arabidopsis thaliana*, the nitrate transporter *NRT1.5* participates in root xylem loading of nitrate (Lin et al., 2008). Peuke (2010) added nitrate to the roots of castor plants (*Ricinus communis* L.) and found that this addition increased the nitrate content of xylem sap, whereas there was almost no nitrate was detected in phloem sap. In this study, the nitrate contents of the basal root woody part (the main transport route was via xylem) and pealed skin (transport was chiefly related to the phloem) on the N+ side increased with the addition of nitrate to the same side and decreased with nitrate withdrawal, indicating that the basal root woody part and pealed skin provide important routes for the transport of nitrate to the shoots.

Parsons et al. (1993) proposed that the roots of all plants must acquire a specific proportion of N supply from the shoots through the phloem. In *A. thaliana* leaves, two nitrate transporters, *NRT1.11* and *NRT1.12*, participate in the translocation of nitrate from the xylem to the phloem for its redistribution to developing leaves (Hsu and Tsay, 2013). Additionally, in *A. thaliana*, the low-affinity nitrate transporter *NRT1.9* is responsible for transporting nitrate through the phloem from the shoots to the roots (Wang and Tsay, 2011). Ohyama and Kawai (1983) used heated steam to girdle the main stems of soybean plants at the pudding stage and found that the nitrate contents of the leaves were basically the same after girdling and without girdling. They therefore thought that nitrate is not easily exported from the soybean leaves through the phloem. In this study, the nitrate contents of the basal root woody part and pealed skin on the N– side increased with the addition of nitrate to the N+ side, indicating that basal root woody part and pealed skin can transport nitrate to the roots. After N withdrawal, the decrease in the nitrate content of the basal root woody part was not significant, and no decrease in the nitrate content of the basal root pealed skin was observed. This phenomenon may be attributed to the nitrate stored in the stems (Ohyama, 1984) and the strict control of N uptake by roots in response to changes in exogenous N availability or in the N demand of the whole plant (Nacry et al., 2013; Vidal et al., 2020), which may have regulated the nitrate contents of the basal root woody part and pealed skin on the N– side.

Mcneil et al. (1979) fed lupin plants with ^14^C-containing asparagine and valine, finding that these two amino acids can be directly translocated from xylem to phloem sap. Asparagine can also be translocated directly from the xylem to the phloem (Pate et al., 1981). The transport of nutrients from plant roots to shoots mainly depends on transpiration by the xylem and can be regulated by the interaction between the xylem and phloem (Krapp et al., 2014). Sulieman et al. (2010) have found that nitrate stimulates asparagine accumulation in the phloem of alfalfa plants, whereas asparagine and glutamine in the phloem sap of soybean seedlings suppress nitrate absorption in soybean roots (Muller and Touraine, 1992). Our study showed that whether on the N+ or the N– side, the nitrate contents of the basal root woody part and pealed skin on the same side exhibited the same trend of changes. Nitrate may be transported in both the basal root woody part and pealed skin, thereby regulating the nitrate balance in soybean plants. However, the specific mechanisms still need to be clarified in future research attempts.

## Conclusion

In the soybean dual-root system, we found that nitrate supplied to one side of the root system was transported to the shoots through the basal root woody part and pealed skin. After distribution by the shoots, nitrate was transported to the roots and nodules on the other side also using the woody part and pealed skin in the basal root, providing the N required for root and nodule growth on the side without N supply.

## Data Availability Statement

The original contributions presented in the study are included in the article/Supplementary Material, further inquiries can be directed to the corresponding author/s.

## Author Contributions

SL and ZG: conceptualization. XL: data curation. CM and ZG: funding acquisition. DY and FX: investigation. SL and XL: methodology. SD and CY: resources. SL: software. SL: writing—original draft. SL and ZG: writing—review and editing. All authors read and agreed to the published version of the manuscript.

## Conflict of Interest

The authors declare that the research was conducted in the absence of any commercial or financial relationships that could be construed as a potential conflict of interest.
