# Supplementary material for: Nitrate Transport and Distribution in Soybean Plants With Dual-Root Systems
Source: Front Plant Sci. 2021 May 20;12:661054. doi: 10.3389/fpls.2021.661054 (PMC8174562; doi:10.3389/fpls.2021.661054)
Supplement: Supplementary file 1 [file Data_Sheet_1.PDF]

S1 Preparation of Plant Materials with a Dual Root System:

Each plastic pot was 0.30 m in diameter and 0.28 m in height and was divided into two equal parts in the middle with a custom-made polycarbonate plastic partition plate. The gap between the plate and the pot was sealed with glue. Two drainage holes 1 cm in diameter were drilled at the bottom of the pot. Each pot was filled with 20 kg of sand.

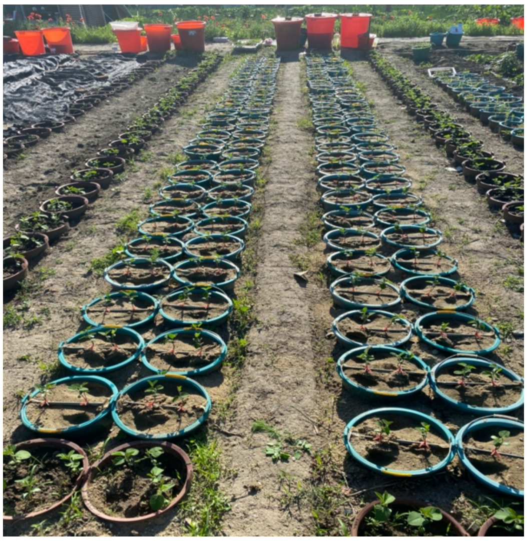

Soybeans sand culture experiments

Dual Root System:

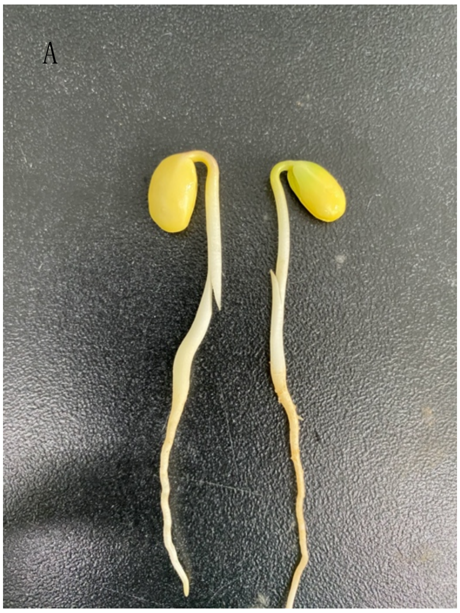


A: The soybean seeds (*Glycine max* L. cv. Dongda ) were seeded into fine-sand medium and cultured in an illuminated growth chamber at 30°C for approximately 3 days. Select soybean embryonic axis with a hypocotyl diameter of 2mm and a total length of 3-6cm, using a sterilization blade to make a 0.5-1.0cm long incision (not cut) up or down at the upper middle of the hypocotyls of the two seedlings.

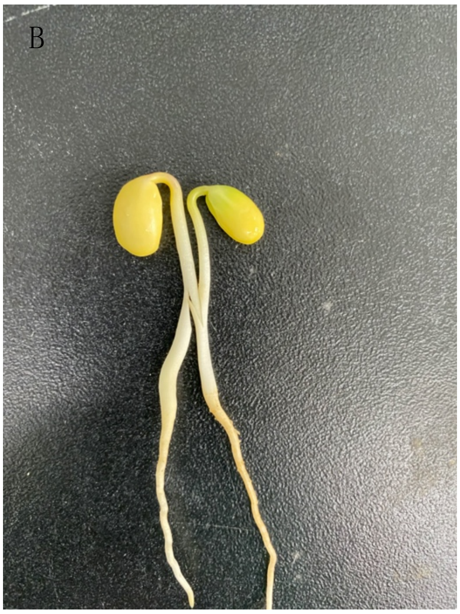


B: Insert the soybean seedlings cut upwards and downwards into each other.

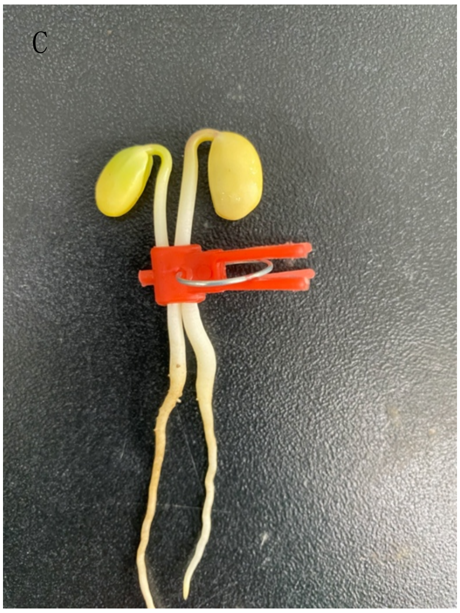


C: Clamp the incision with grafting clip.

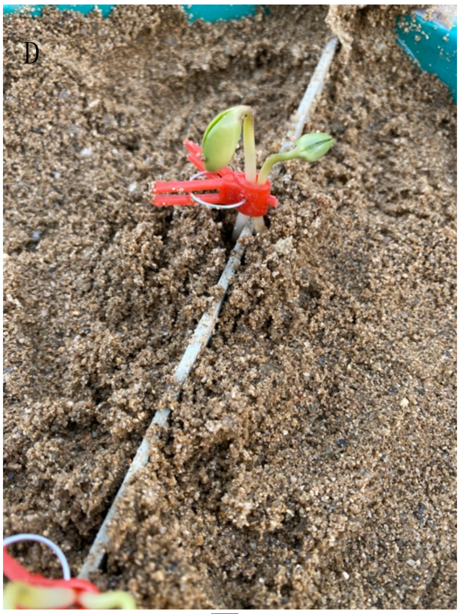


D: The roots of the two seedlings were planted into fine-sand medium of each half of the pot divided by the partition, and the grafting site was just above the partition plate.

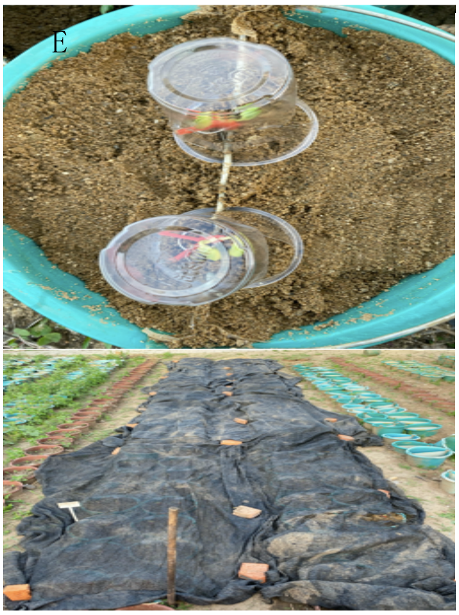


E: Dual root seedlings were fastened with transparent plastic cups with vents and covered with shading net to prevent the effects of rain and strong light on grafted seedlings.

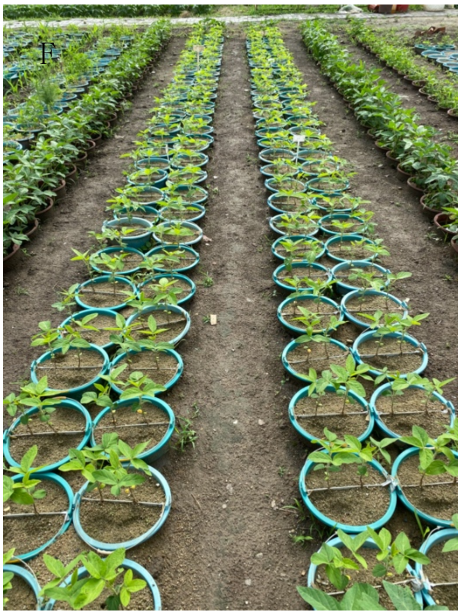


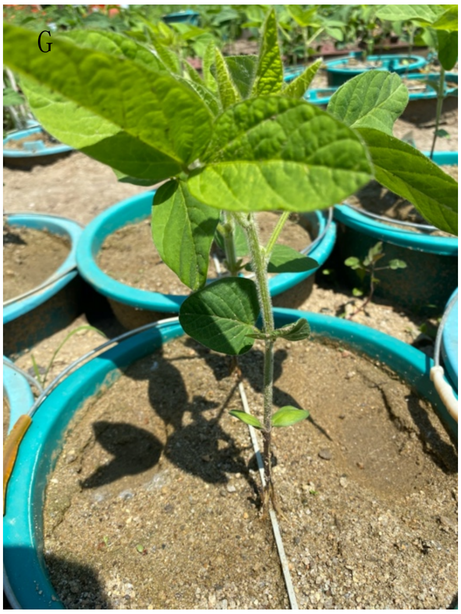


F-H: Remove the sunshade net, plastic cup and grafting clip after one week. Cut off one shoot from the grafting, so that the seedling becomes a whole with two roots sharing the same shoot.

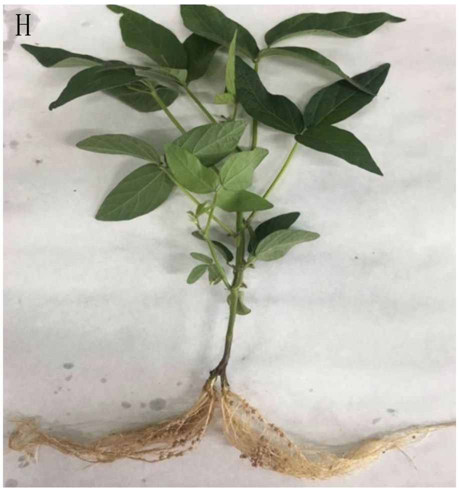

**S2 Concentration of N-free nutrient solution of sand culture**

| Inorganic salts | Concentration (mg/L) | Inorganic salts | Concentration (mg/L) |
|---|---|---|---|
| $KH_2PO_4$ | 136.00 | $ZnSO_4 \cdot 7H_2O$ | 0.22 |
| $MgSO_4$ | 240.00 | $MnCl_2 \cdot 4H_2O$ | 4.90 |
| $CaCl_2$ | 220.00 | $H_3BO_3$ | 2.86 |
| $Na_2MoO_4 \cdot H_2O$ | 0.03 | Fe–EDTA* | |
| $CuSO_4 \cdot 5H_2O$ | 0.08 | | |

*Note: The solution which contain 5.57 g $FeSO_4 \cdot 7H_2O$ and 7.45 g $Na_2EDTA$ per one L respectively were added into nutrient medium as the rate of 1:1000 when using.

S3 method of base root sampling in nitrate concentration change test base.

Dual-root system was schematized in figure 1A whence nitrate concentration change test was sampled from the red frame area. Figure 1B: schematic of root dissection under the optical microscope, the double roots were severed at 2 cm from the grafting, followed by peeling the outer skin, including epidermis, cortex and phloem, the main transport channel is the phloem, noted as phloem$^+$, while the rest including xylem and pith, noted as xylem$^+$ .

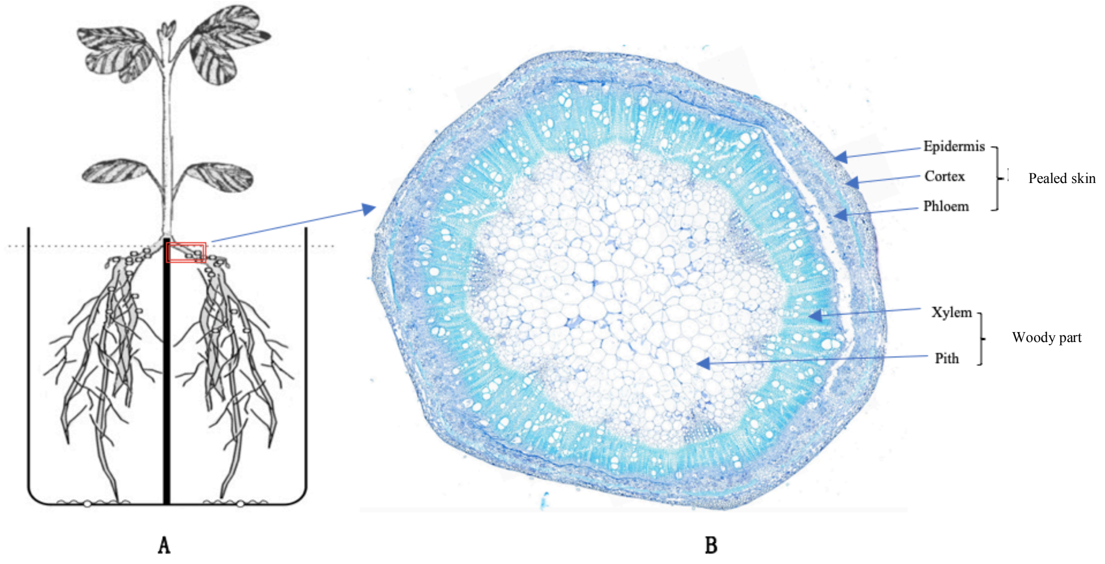


S4 Dry weight of soybean nodules in nitrate content change experiment (g plant$^{-1}$)

| Treatments | Nitrogen concentration (mg·L$^{-1}$) | Phase I | | Phase II | | Phase III | |
|---|---|---|---|---|---|---|---|
| | | N- | N+ | N- | N+ | N- | N+ |
| $N_{TTT}$ | 0-0-0 | 1.16±0.05 a | 1.16±0.05 a | 1.25±0.10 a | 1.25±0.10 a | 1.34±0.12 a | 1.34±0.12 a |
| $N_{HHH}$ | 100-100-100 | 1.25±0.22 a | 1.17±0.09 a | 1.22±0.04 a | 1.07±0.19 a | 1.16±0.21 a | 1.11±0.04 bc |
| $N_{HTT}$ | 100-0-0 | | | 1.19±0.06 a | 1.07±0.08 a | 1.30±0.31 a | 1.24±0.02 ab |
| $N_{HTH}$ | 100-0-100 | | | | | 1.08±0.21 a | 1.00±0.02 c |

Note: The treatments and N concentrations are described in Table 1. Values are means ± standard error (n=3). Different lowercase letters indicate a significant difference between the treatments at the 5% level (Duncan's test) by longitudinal comparison.

S5 Nitrogenase activity of soybean nodules in nitrate content change experiment （$C_2H_4$ μmol $h^{-1}$ $plant^{-1}$）

| Treatments | Nitrogen concentration ($mg \cdot L^{-1}$) | Phase I | | Phase II | | Phase III | |
|---|---|---|---|---|---|---|---|
| | | N- | N+ | N- | N+ | N- | N+ |
| $N_{TTT}$ | 0-0-0 | 55.84±1.59 a | 55.84±1.59 a | 37.34±1.47 a | 37.34±1.47 a | 95.25±0.83 a | 95.25±0.83 a |
| $N_{HHH}$ | 100-100-100 | 38.37±1.04 b | 12.01±1.57 b | 26.29±0.14 b | 16.11±1.80 c | 37.96±1.12 c | 25.32±1.08 c |
| $N_{HTT}$ | 100-0-0 | | | 28.40±1.96 b | 24.20±0.78 b | 54.48±1.51 b | 36.31±2.40 b |
| $N_{HTH}$ | 100-0-100 | | | | | 34.86±1.05 c | 11.18±0.68 d |

Note: The treatments and N concentrations are described in Table 1. Values are means ± standard error (n=3). Different lowercase letters indicate a significant difference between the treatments at the 5% level (Duncan's test) by longitudinal comparison.
